# Human Rhinovirus B and C Genomes from Rural Coastal Kenya

**DOI:** 10.1128/genomeA.00751-16

**Published:** 2016-07-28

**Authors:** Charles N. Agoti, Patience K. Kiyuka, Everlyn Kamau, Patrick K. Munywoki, Anne Bett, Lia van der Hoek, Paul Kellam, D. James Nokes, Matthew Cotten

**Affiliations:** a Epidemiology and Demography Department, KEMRI—Wellcome Trust Research Programme, Kilifi, Kenya; b School of Health and Human Sciences, Pwani University, Kilifi, Kenya; c Laboratory of Experimental Virology, Academic Medical Center of the University of Amsterdam, Amsterdam, The Netherlands; d Imperial College London, London, United Kingdom; e KyMab Limited, Cambridge, United Kingdom; f School of Life Sciences and WIDER, University of Warwick, Coventry, United Kingdom; g The Wellcome Trust Sanger Institute, Cambridge, United Kingdom

## Abstract

Primer-independent agnostic deep sequencing was used to generate three human rhinovirus (HRV) B genomes and one HRV C genome from samples collected in a household respiratory survey in rural coastal Kenya. The study provides the first rhinovirus genomes from Kenya and will help improve the sensitivity of local molecular diagnostics.

## GENOME ANNOUNCEMENT

Human rhinoviruses (HRVs) are nonenveloped viruses with a single-stranded positive-sense RNA genome of approximately 7,200 nucleotides with a single open reading frame (ORF) encoding a polyprotein that is cleaved into four structural proteins and seven nonstructural proteins (1). These viruses belong to the *Enterovirus* genus within the *Picornaviridae* family, with three species currently defined (HRV-A, HRV-B, and HRV-C) (2). In addition to being widely recognized as the major cause of common cold syndrome, HRVs have been increasingly diagnosed in individuals with lower respiratory tract infections (3), although underlying causality remains uncertain (3–5).

Typical of RNA viruses, HRVs can evolve rapidly due to an error-prone RNA-dependent RNA polymerase, short-generation times, and large population sizes. To maintain the sensitivity of clinical molecular diagnostics for these viruses, it is essential that diagnostic primers recognize currently circulating viruses. It is therefore useful to routinely sequence a subset of clinical samples using unbiased, random priming methods combined with *de novo* assembly genome methods (6). The resulting sequence data provide a less biased description of the circulating viruses in a community, and the approach has the potential to reveal viruses that diagnostic primers fail to detect. As part of a household cohort study undertaken in Kilifi county, coastal Kenya, samples were collected from every household member twice weekly, independent of symptoms (7). As a by-product of an agnostic sequencing of coronavirus positive samples detected in the survey, we generated four nearly complete rhinovirus genomes, three HRV-B and one HRV-C, which are reported here. These data represent the first HRV full genomes from Kenya.

Phylogenetic analysis of the VP4-2 junctions of each viral genome compared with reference sequences revealed that the HRV-B viruses belonged to type HRV-B37 (*n* = 1) and HRV-B48 (*n* = 2), while the single HRV-C genome was assigned to HRV-C35 (2). The two HRV-B48 viruses were identical in the VP4-2 region, but four nucleotide differences were noted elsewhere in their genomes.

Of interest, the real-time reverse transcriptase PCR primers used in the local viral diagnostic lab (8) were found to have nucleotide differences from the HRV-B genomes reported here. Several mismatches to both the forward and reverse primers are predicted to compromise PCR function. In fact, the three samples yielding HRV-B genomes were rhinovirus-negative by the diagnostic assay. It is noteworthy that HRV-B was identified at very low prevalence in this household cohort (unpublished data) and the samples yielding HRV-B genomes were classified as negative by this PCR assay. While it was previously reported that HRV-B circulates at lower prevalence than either HRV-A or C (3, 9), it cannot be excluded that a contributing factor to the low HRV-B prevalence was the diagnostic assay failing to detect some of the locally circulating HRV strains. The availability of local full genomes generated using a primer-independent method provides an important tool for improving diagnostic methods.

### Nucleotide sequence accession numbers.

The complete genome sequences of the rhinovirus genomes are available at GenBank under the accession numbers KX348029 to KX348032.
